# Pd–Cu Single-Atom Catalysts in Covalent Organic Frameworks for Sonogashira Cross-Coupling Reaction

**DOI:** 10.1021/acs.chemmater.6c00391

**Published:** 2026-06-24

**Authors:** Murad Najafov, Daniele Bonavia, Felipe Gándara, Maarten Nachtegaal, Mounir Mensi, Ali Coskun

**Affiliations:** † Department of Chemistry, 27211 University of Fribourg, Chemin du Musee 9, Fribourg 1700, Switzerland; ‡ National Centre of Competence in Research (NCCR) Catalysis, University of Fribourg, Fribourg 1700, Switzerland; § Center for Photon Science, 28498 PSI, Villigen CH-5232, Switzerland; ∥ Materials Science Institute of Madrid—CSIC, Sor Juana Inés de la Cruz 3, Madrid 28049, Spain; ⊥ Center for Energy and Environmental Sciences, PSI, Villigen CH-5232, Switzerland; # Department of Chemistry, ETH Zürich, Zürich CH-8093, Switzerland; ∇ Institute of Chemical Sciences and Engineering (ISIC), 150727 École Polytechnique Fédérale de Lausanne, Sion 1950, Switzerland

## Abstract

Bimetallic single-atom catalysts containing two distinct metals within well-defined and spatially controlled environments can offer cooperative reactivity. Here, we present a versatile design strategy for constructing metallophthalocyanine covalent organic frameworks (COFs) containing precisely engineered Pd and Cu sites through mixed-metal ionothermal synthesis, followed by wet impregnation. The corresponding COF, named Pd–Cu@pyPPC–NaCl, is synthesized through the polymerization of tetracyanopyrazine in a molten salt mixture of CuCl_2_/ZnCl_2_/NaCl, enabling the incorporation of Cu within the N_4_ phthalocyanine cavity, followed by the incorporation of Pd as the secondary metal into the pore through wet impregnation. This orthogonal metal incorporation yields atomically dispersed and spatially controlled catalytic centers with Cu and Pd loadings of 16.2 and 6.2 wt %, respectively, in a crystalline organic support. Pd–Cu@pyPPC–NaCl exhibits excellent catalytic performance in the Sonogashira–Hagihara cross-coupling of iodobenzene and phenylacetylene, delivering yields above 95% in batch and above 90% under continuous-flow conditions with good selectivity. The significantly lower catalytic activity of control COFs involving Pd@pyPPC–NaCl and Cu@pyPPC–NaCl supported synergistic catalytic activity, in which Pd and Cu fulfill complementary roles in aryl halide activation and alkyne activation, respectively, on the COF support.

## Graphical abstract

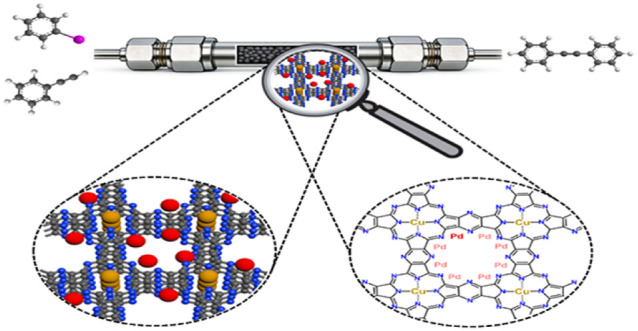

## Introduction

The rational design of heterogeneous single-atom catalysts (SACs) that integrate spatially controlled two distinct metal sites within a single, structurally ordered framework remains a major challenge in catalysis. Bimetallic systems can display cooperative effects that influence electronic structure, adsorption energetics, and reaction pathways, thereby leading to enhanced activity, selectivity, and stability compared to monometallic analogues.^1–3^ Achieving such cooperative behavior, however, requires a high degree of control over the position and coordination environment of metal ions. In many cases, traditional routes such as co-reduction,^4^ impregnation on disordered supports,^5^ or galvanic exchange^6^ do not offer precise control over either the coordination environment or the position of metal ions and instead give rise to statistically distributed or partially segregated metal domains.^7^ Accordingly, synthetic strategies that allow two different metals to be positioned in a predictable and site-defined manner within crystalline architectures are essential for establishing a clear structure–property relationship in bimetallic SACs.^8^ Covalent organic frameworks (COFs) provide an ideal platform to address this challenge owing to their tunable porosity and modular design.^9^ Their periodic arrangement of functional sites allows the precise immobilization of metal species in predictable geometries.^10^ Among these materials, metallophthalocyanine-based COFs are particularly attractive due to the highly stable N_4_ macrocyclic cavities of the phthalocyanine units, which can effectively coordinate a broad range of transition metals.^11^ Notably, the planar π-conjugated macrocycles facilitate charge delocalization across stacked layers,^12^ and enable electronic communication between adjacent metal centers, which results in improved catalytic performance.^13–16^ More recently, several COFs have been employed in electrocatalysis,^15^ photocatalysis,^17^ and organic transformations, including CO_2_ reduction^2^ and cross-coupling reactions,^18^ demonstrating their versatility as tunable catalytic platforms. However, the reported systems have almost exclusively relied on monometallic compositions with uniform coordination environments. In this direction, we reasoned that a mixed-metal ionothermal synthesis approach can be adopted to form bimetallic COFs with precisely controlled coordination environment and spatial arrangement. In this architecture, one metal center is primarily anchored within the phthalocyanine cavity, whereas the second metal is incorporated into the pores, where it can modulate the electronic structure of the framework or directly participate in the substrate activation. Such dual-site catalysts can facilitate sequential reaction steps,^19^ similar to those observed in enzymatic systems.^20,21^ In single-atom and mixed-metal heterogeneous catalysts, differences in coordination preferences between the metal species and the support can lead to competition between metal–support interactions and metal–metal bonding.^22^ During the synthesis or under operating conditions, this competition may cause structural changes such as metal aggregation into clusters or alloys,^23^ partial amorphization of the support,^24^ or nonuniform active site environments.^25^ These effects hinder the ability of the catalyst to keep metal atoms isolated and the active sites uniform. As a result, coordination compatibility between the metals and the support becomes essential. This is particularly challenging to realize in bimetallic SACs and in systems with high metal loadings.

To overcome these limitations, here, we employed (Figure 1) a mixed-metal ionothermal synthesis strategy that enables COF formation using tetracyanopyrazine in a molten-salt mixture of CuCl_2_/ZnCl_2_/NaCl, in which ZnCl_2_ serves both as a solvent and catalyst for the polymer formation.^11^ In addition, despite having excess ZnCl_2_, higher complex stability of Cu­(II) facilitates its dynamic incorporation into the N_4_ macrocyclic cavities of the phthalocyanine units. Targeted integration of Cu ions into the phthalocyanine cavity enables precise integration of a range of secondary metal ions into the pores. As such, the subsequent introduction of Pd­(II) ions into the COF pores was achieved through controlled wet impregnation, leading to the formation of bimetallic COF, named Pd–Cu@pyPPC–NaCl with Cu and Pd loadings of 16.2 and 6.2 wt %, respectively. XPS and EXAFS analyses revealed that the COF structure primarily contains Pd­(II) ions and Cu­(II) metal ions, along with a small amount of Cu­(I), which is presumably formed during the formation of the polymer network. To demonstrate the synergistic effect between the two metal sites, we also synthesized two control COFs containing either Pd or Cu ions in both cavity and pore, Pd@pyPPC–NaCl (22.2 wt %) and Cu@pyPPC–NaCl (16.5 wt %), respectively. The complementary roles of Cu and Pd in alkyne activation and transmetalation are consistent with the observed significantly enhanced catalytic performance compared to the single metal-loaded control COFs in the Sonogashira–Hagihara cross-coupling reaction, delivering excellent yields above 95% in batch and above 90% under continuous-flow conditions with good selectivity under both batch and continuous flow conditions.

**Figure 1:**
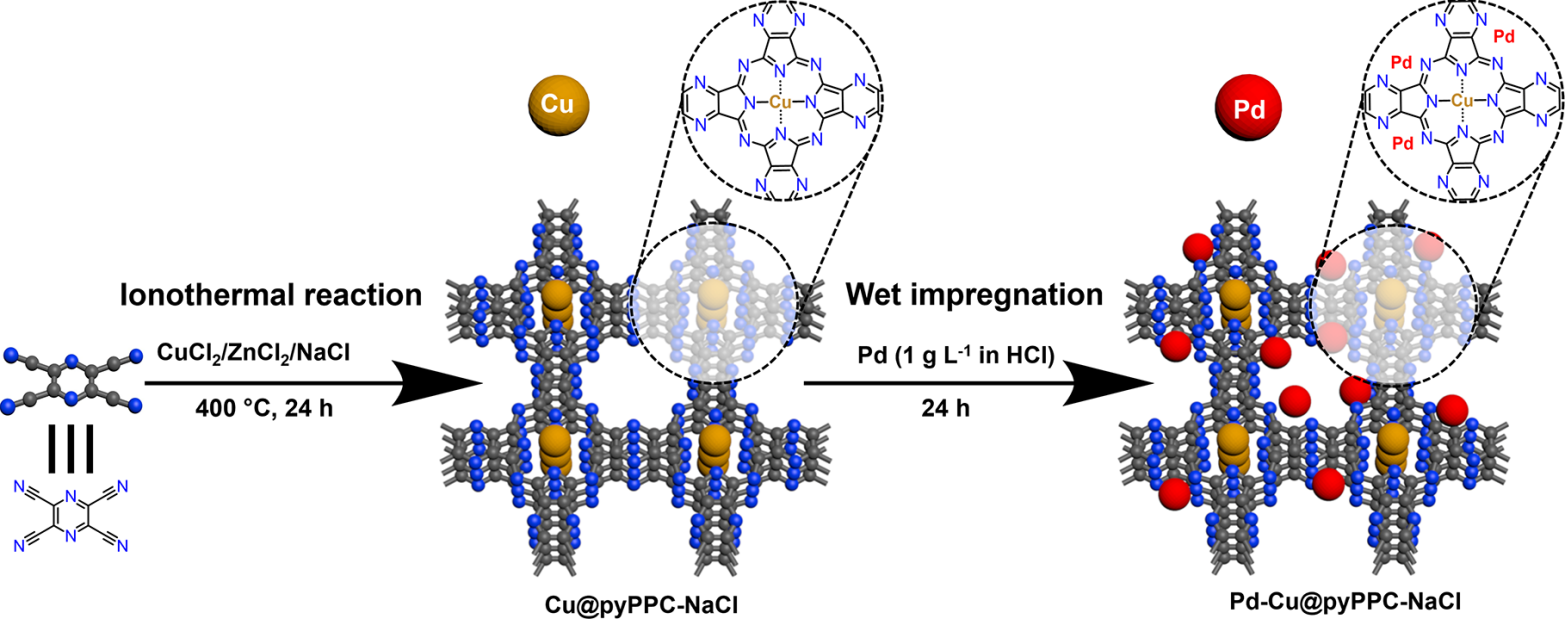
Schematic illustration of the stepwise synthesis of the bimetallic Pd–Cu@pyPPC–NaCl COF, with cavity-bound Cu sites formed under ionothermal conditions, followed by the incorporation of Pd into the pores through wet impregnation.

## Results and Discussion

Mixed-metal ionothermal synthesis is a powerful approach to introduce various metal ions into the polymer networks, for which the Irving-Williams series offers a guideline to the metal pairs that can be used. Whereas the use of ZnCl_2_ is well established in the context of ionothermal synthesis owing to its relatively low melting point of 290 °C, which can be further reduced by forming a eutectic salt mixture with NaCl, the direct use of metal salts such CuCl_2_, NiCl_2_, PdCl_2_ is not possible due to their high melting points above 500 °C. The fact that ZnCl_2_ serves both as a solvent and catalyst, the addition of a secondary metal ion to ZnCl_2_ enables its direct incorporation into the framework, provided that its complex stability is higher than that of Zn­(II). In the Irving-Williams complex stability order, Cu­(II) shows much higher stability compared to that of Zn­(II), thus facilitating the incorporation of Cu­(II) into the N_4_ macrocyclic cavity of phthalocyanine unit within the COF. Furthermore, the availability of pores in the COF structure opens the possibility of integration of various secondary metal ions. Accordingly, we synthesized three COF structures using mixed-metal ionothermal approach. The polymerization of tetracyanopyrazine under mixed-metal ionothermal conditions using the salt mixture of CuCl_2_/ZnCl_2_/NaCl at 400 °C enabled the formation of Cu@pyPPC–NaCl with a Cu content of 16.5 wt %. The removal of Cu­(II) from the pores and subsequent integration of Pd­(II) through wet-impregnation enabled the synthesis Pd–Cu@pyPPC–NaCl with Pd and Cu contents of 6.2 and 16.2 wt %, respectively. Notably, this approach serves as a predefined coordination site for bimetallic COFs for a primary metal (Cu), while the nitrogen-rich pore network provides a secondary (Pd) coordination domain. In addition, we also synthesized Pd@pyPPC–NaCl with a Pd content of 22.2 wt % using the salt mixture of PdCl_2_/ZnCl_2_/NaCl under the same conditions.^26^

Powder X-ray diffraction (PXRD) patterns of Cu@pyPPC–NaCl and Pd–Cu@pyPPC–NaCl are shown in Figure 2a (Tables S1 and S2). Both samples display two broad reflections centered at 2θ ≈ 8.8° and 28°, consistent with the formation of layered 2D COFs, and similar to previously reported phthalocyanine-based conjugated frameworks.^27,28^ The patterns are in agreement with **sql** layers, as imposed by the geometry and connectivity of the pyrazinophthalocyanine building unit. The idealized structural model adopts the *P4/mmm* space group, with optimized lattice parameters a = 10.48 Å and c = 3.42 Å. Within this model, the low-angle reflection is assigned to the (100) plane, whereas the broad peak at 2θ ≈ 28° is dominated by the (001) reflection, with a contribution from (101). The pronounced peak broadening indicates limited crystallite size and/or partial stacking order, a behavior commonly observed for ionothermally synthesized phthalocyanine-based polymers.^11^ Notably, the Cu@pyPPC–NaCl and Pd–Cu@pyPPC–NaCl patterns are essentially identical, suggesting that incorporation of Pd ions does not disturb long-range periodicity; instead, Pd species are likely incorporated by adopting multiple symmetry-equivalent binding configurations within the pores (up to eight per pore) without a translationally ordered arrangement along the layers. Consistently, the simulated PXRD pattern for a structural model with partially occupied palladium and chlorine sites reproduces the main experimental features. The lack of additional diffraction lines in the PXRD pattern of Pd–Cu@pyPPC–NaCl is supportive of this proposed distribution of Pd atoms along the layers. No additional reflections associated with metal nanoparticles or metal oxide phases are observed, suggesting that metal ions are atomically dispersed within the polymer matrix rather than forming nanoparticles. This observation agrees with previous studies on metal–phthalocyanine frameworks, where the metal ions remain uniformly coordinated to nitrogen centers within the macrocyclic network.^29–35^ Inductively coupled plasma-optical emission spectrometry (ICP-OES) analysis was performed to determine the metal contents of the synthesized COFs, and the results are summarized in Table S3. The analysis showed Cu loading of 16.5 wt % for Cu@pyPPC–NaCl. Elemental analysis (EA) results (Table S4) provided the C, H, and N contents, and were found to be consistent with the expected framework compositions. Thermogravimetric analysis (TGA) profiles of Cu@pyPPC–NaCl (Figure S1) showed a small weight loss below 200 °C, corresponding to the removal of adsorbed or residual solvent molecules, and thermal stability up to 400 °C under air. The residual mass observed at elevated temperatures corresponds to the presence of metal species within the polymer matrix. The formation of phthalocyanine moieties within the polymer network was verified by Fourier transform infrared (FT-IR) spectroscopy (Figure 2b). In the spectrum of Cu@pyPPC–NaCl, the complete disappearance of the nitrile (−C≡N) stretching vibration at approximately 2250 cm^–1^ confirms successful cyclotetramerization of the tetracyanopyrazine precursors, a feature consistently observed for all the polymers. Additional bands observed at around 1237 cm^–1^ and 1092 cm^–1^ are attributed to C–N stretching vibrations of the pyrazine units that link neighboring phthalocyanine macrocycles. A characteristic absorption near 890 cm^–1^ is assigned to metal–nitrogen (M–N) coordination within the phthalocyanine core. Together, these spectral features demonstrate the successful formation of a monometallic COF structure stabilized by strong M–N coordination bonds. Secondary metal incorporation was carried out postsynthetically via a wet-impregnation approach, in which an aqueous PdCl_2_ (9.4 mM, corresponding to 1 g L^–1^ Pd in HCl) solution was applied in excess relative to the pore volume of the Cu@pyPPC–NaCl framework, enabling the introduction of Pd­(II) into the pores of the COF structure.^36,37^ The use of excess PdCl_2_ ensures complete wetting of the framework and access to the nitrogen-rich coordination sites within the phthalocyanine network. During impregnation, diffusing Pd ions interact with these sites, leading to controlled incorporation without detectable disruption of the framework structure. Inductively coupled plasma analysis revealed (Table S3) Cu content decreasing slightly from 16.5 wt % to 16.2 wt % and Pd content of 6.2 wt %, indicating the exchange of residual Cu­(II) located within the pores with the Pd ions.

**Figure 2:**
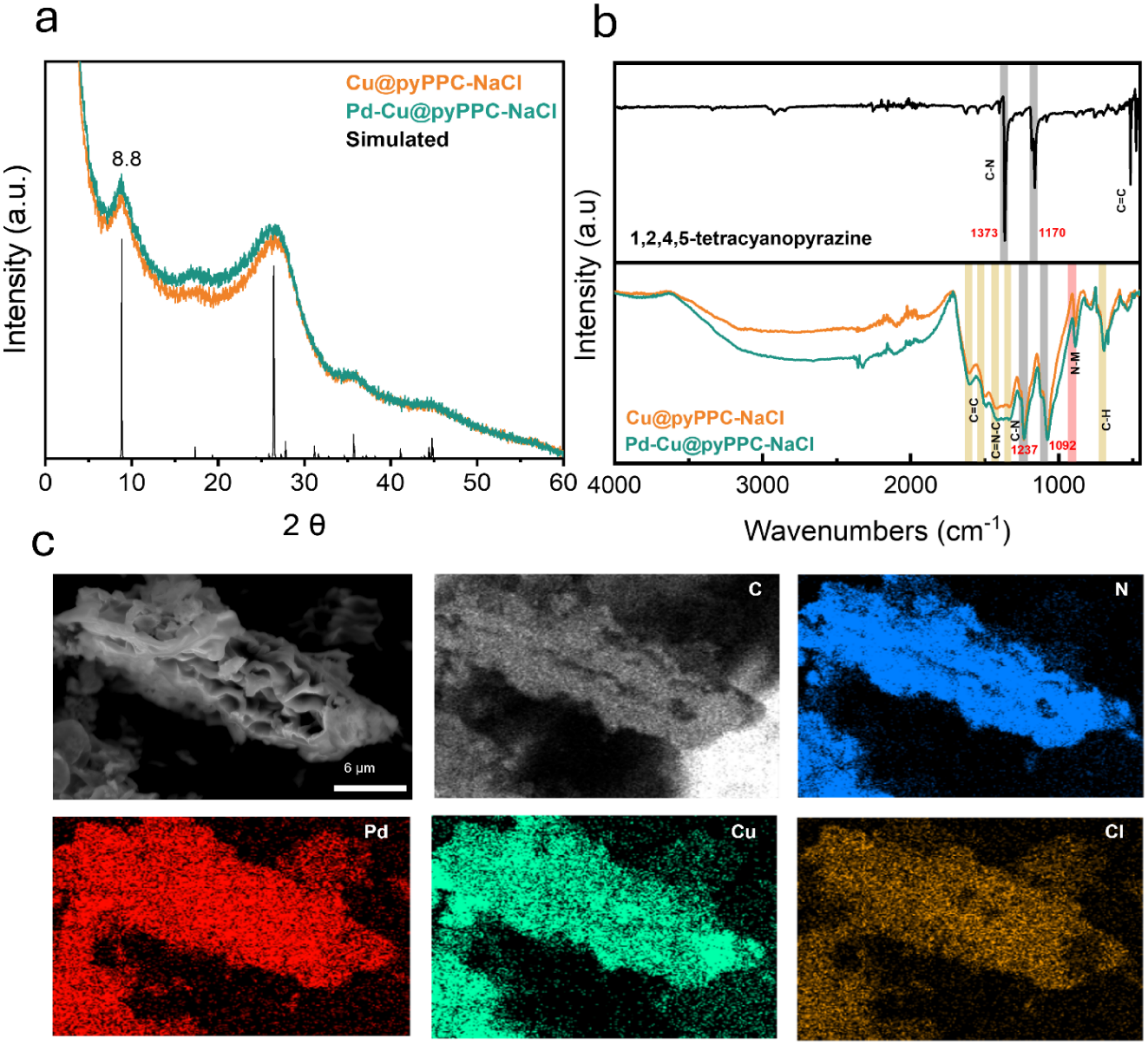
Structural and elemental characterization of the Cu@pyPPC–NaCl and Pd–Cu@pyPPC–NaCl COF. (a) Experimental and simulated powder X-ray diffraction (PXRD) patterns of Cu@pyPPC–NaCl and Pd–Cu@pyPPC–NaCl (b) FTIR spectra of Cu@pyPPC–NaCl and Pd–Cu@pyPPC–NaCl, (c) SEM image and EDX elemental mappings (C, N, Pd, Cu, Cl) of Pd–Cu@pyPPC–NaCl.

The surface morphology and metal distribution of the polymers were examined by scanning electron microscopy (SEM) and energy-dispersive X-ray spectroscopy (EDX), as shown in Figure 2c. The Pd–Cu@pyPPC–NaCl showed a similar morphology consisting of irregularly stacked and aggregated sheet-like particles, which is typical for phthalocyanine-based polymers synthesized under ionothermal conditions. The layered structure indicates the formation of a conjugated two-dimensional framework.^14,38^ The EDX mapping of the Pd–Cu@pyPPC–NaCl polymer revealed a uniform distribution of Cu and Pd ions throughout the sample without any visible sign of particle segregation or metal-rich domains. This homogeneous bimetallic dispersion confirms that both metal centers were successfully incorporated into the polymer matrix. Such uniform and layered structures are favorable for maintaining accessible sites and enhancing diffusion during catalytic applications.^39,40^

Brunauer–Emmett–Teller (BET) surface area analysis was conducted using N_2_ adsorption isotherms at 77 K to evaluate the porosity of Cu@pyPPC–NaCl (Figure S2). The BET surface area was determined to be 480 m^2^ g^–1^ (Table S2). The adsorption isotherm displays predominantly Type I behavior, indicating the formation of a microporous network. To determine the pore size distribution of the polymer, nonlocal density functional theory (NLDFT) analysis was applied (Carbon–N_2_ at 77 K, heterogeneous surface model). The pore size distribution analysis reveals two distinct pore populations, with an ultramicroporous contribution centered at approximately 0.59 nm and a microporous contribution in the range below 1.6 nm, as shown in Figure S2d.

X-ray photoelectron spectroscopy (XPS) was used to examine molecular connectivity along with the incorporation and electronic state of Pd and Cu atoms within Pd–Cu@pyPPC–NaCl (Figure 3). The survey spectrum shows signals from C, O, N, Cu, Pd, and Cl only, which matches the target COF composition. In the high-resolution spectra, the C 1s region is dominated by aromatic C–C/C=C and C–N contributions, consistent with an extended conjugated backbone and a nitrogen-rich framework. The N 1s spectrum contains two components assigned to aza-bridging and pyrrolic nitrogen atoms, which represent the characteristic coordination environments of the phthalocyanine structure and serve as binding sites for the metal centers. The Pd 3d region displays a clear doublet at 337.3 and 342.5 eV, characteristic of Pd­(II) in a coordination-stabilized, positively charged state. The fact that no features associated with metallic Pd appear in the spectrum, it excludes the presence of Pd nanoparticles and points to atomically dispersed Pd sites within the framework. Similarly, the Cu 2p spectrum shows features characteristic of Cu­(II), including the expected satellite peaks, which indicate that Cu remains in an oxidized state coordinated to the framework. We also observed a small amount of Cu­(I) species, presumably due to the partial reduction of Cu­(II) during the formation of the framework structure. X-ray absorption spectroscopy (XAS) was used to elucidate the local coordination environments and oxidation states of Cu and Pd in the bimetallic Pd–Cu@pyPPC–NaCl framework. The Cu and Pd K-edge X-ray absorption near-edge structure (XANES) spectra display edge positions and white-line intensities characteristic of Cu­(II) and Pd­(II), respectively, with no features associated with metallic Cu(0) or Pd(0) species (Figure 3f,g). In the Cu K-edge XANES spectra, features observed in the pre-edge region are assigned to 1s → 3d transition and a weak 1s → 4p rising edge transition, which are diagnostic of a Cu^2+^ (3d^9^) electronic configuration and absent for Cu^+^ species due to their filled 3d^10^ shell. The presence of this pre-edge feature, therefore, indicates that Cu is predominantly present as Cu^2+^ in the bulk of the material, as also verified by its overlap with CuO. As stated previously, XPS analysis of the Cu 2p region shows Cu^2+^ as the dominant surface species, with characteristic shakeup satellites, while suggesting a minor Cu^+^ contribution. The Pd K-edge XANES closely matches that of PdO and H_2_PdCl_4_, supporting the presence of isolated Pd^2+^ centers rather than aggregated clusters. Fourier-transformed extended X-ray absorption fine structure (EXAFS) analysis further supports the proposed local coordination environment (Figure S3). The FT Cu K-edge EXAFS spectra are best fitted by a single Cu–N coordination shell, yielding an average coordination number of 3.8 ± 0.8 and a slight contraction of the Cu–N bond distance relative to the ideal phthalocyanine environment (Table S5). No Cu–Cu contributions are detected in either k or R space, suggesting the absence of Cu clustering.

**Figure 3:**
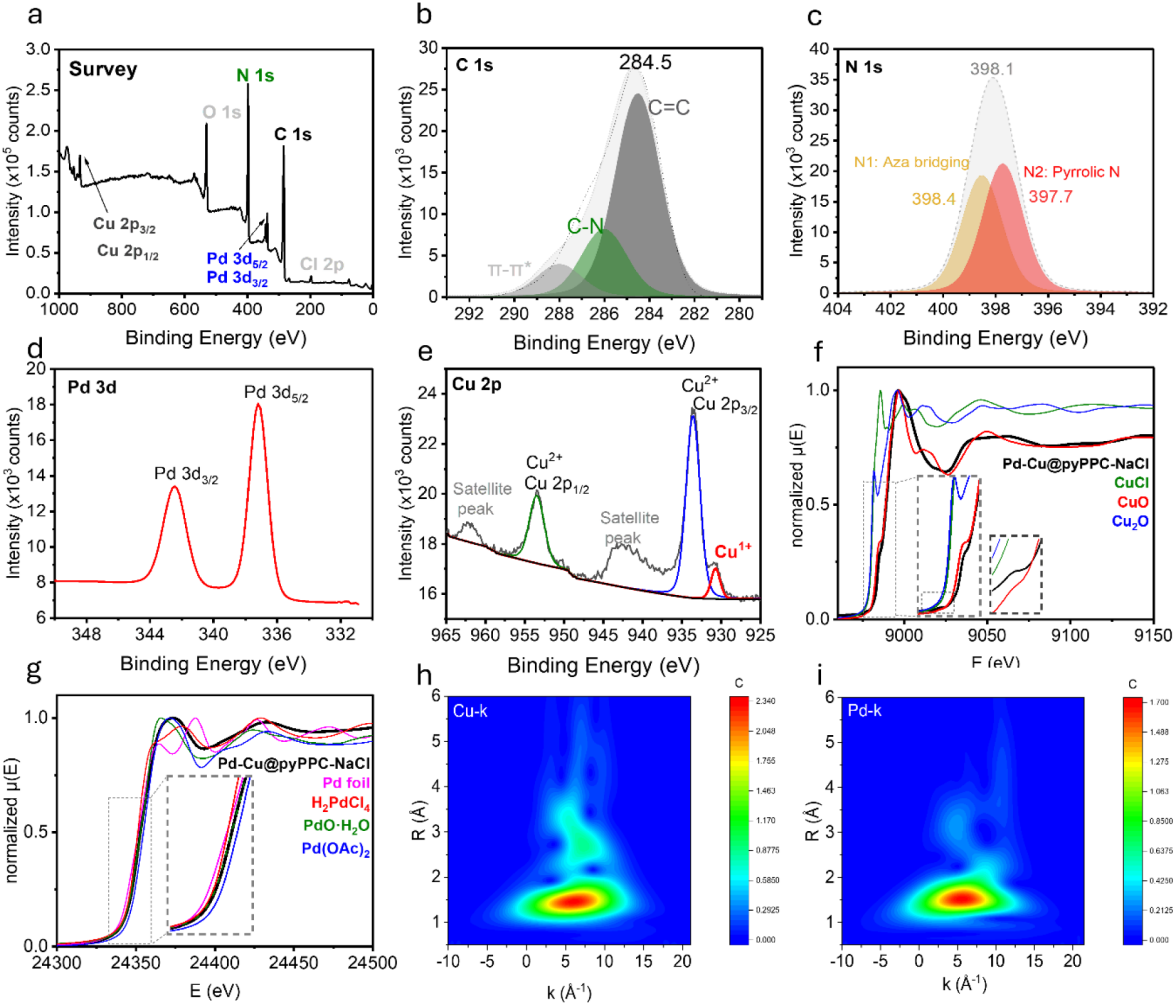
X-ray photoelectron spectroscopy (XPS) analysis of Pd–Cu@pyPPC–NaCl. (a) XPS survey spectrum confirming the presence of C, N, Cu, Pd, O and Cl elements. (b) High-resolution C 1s spectrum deconvoluted into C–C/C=C, C–N, and minor oxidized carbon contributions. (c) High-resolution N 1s spectrum showing contributions from aza-bridging nitrogen and pyridinic nitrogen species. (d) High-resolution Pd 3d spectrum with Pd 3d_5_/_2_ and Pd 3d_3_/_2_ peaks characteristic of positively charged Pd species. (e) High-resolution Cu 2p spectrum exhibiting Cu 2p_3_/_2_ and Cu 2p_1_/_2_ features with satellite peaks. (f) Normalized Cu K-edge XANES spectra of Pd–Cu@pyPPC–NaCl and Cu reference compounds, consistent with a Cu^+^ coordination environment. (g) Normalized Pd K-edge XANES spectra of Pd–Cu@pyPPC–NaCl compared with Pd foil and Pd reference compounds, revealing an edge position and spectral features distinct from metallic Pd. (h) Wavelet-transform (WT) contour plot of Cu K-edge EXAFS, showing a dominant Cu–N coordination environment without Cu–Cu contributions. (i) Wavelet-transform (WT) contour plot of Pd K-edge EXAFS, confirming the absence of Pd–Pd scattering paths and validating the dispersion of Pd.

At the Pd K-edge, the FT EXAFS spectra are dominated by a broad first-shell contribution arising from coordination to light scatterers, with no evidence of Pd–Pd scattering, consistent with the formation of atomically dispersed Pd sites (Figure S3). Because the Pd–N and Pd–Cl contributions occur at similar distances and involve low-Z scatterers, they cannot be reliably separated and were therefore treated as a single effective coordination shell with an overall coordination number of 2.7 ± 0.4 (Table S6), highlighting the intrinsic limitations of EXAFS for resolving closely spaced light-atom coordination environments in this system. At R ≈ 3 Å, the EXAFS signal is dominated (Figure 3h and 3i) by first-shell metal–N/C scattering, with no features observed at higher radial distances. This observation is further supported by wavelet transform analysis of both Cu and Pd K-edge spectra shows a single intensity maximum at low k and R ≈ 2–3 Å, while high-k contributions characteristic of metal–metal scattering is absent, confirming atomic dispersion of the metal sites.

The catalytic performance of the Pd–Cu@pyPPC–NaCl was comparatively investigated with Pd@pyPPC–NaCl and Cu@pyPPC–NaCl using the Sonogashira–Hagihara cross-coupling reaction of iodobenzene and phenylacetylene as a benchmark reaction (Figure 4a). This reaction was selected because it requires cooperative activation of both the aryl halide and the terminal alkyne and thus probes Pd–Cu synergy within the covalent organic framework. In Pd–Cu bimetallic COF, Pd serves as the main catalytic center for the oxidative addition of the aryl halide, while Cu assists in activating the alkyne and promoting transmetalation.^41^ These cooperative interactions are further enabled by the structural characteristics of the material: the high specific surface area and porosity promote efficient mass transport and substrate accessibility, while the high density of atomically dispersed metal sites ensures a maximized population of catalytically active centers. The proximity of Pd and Cu sites within the conjugated network could facilitate the complementary roles of the two metals during the catalytic cycle, thus truly mimicking their homogeneous counterparts. We systematically evaluated the influence of base and solvent on the catalytic performance to optimize the reaction conditions. Initially, we screen different bases, including Cs_2_CO_3_, K_2_CO_3_, Na_2_CO_3_, KOAc, KOH, DIPEA, and NEt_3_ using Pd–Cu@pyPPC–NaCl in DMSO at 100 °C for 16 h (Figure 4b and Table S7). Among the tested bases, potassium acetate (KOAc) gave the highest yield (86%), while potassium hydroxide (KOH) also showed good activity (64%), likely due to its strong basicity promoting rapid alkyne activation, albeit with reduced selectivity. In contrast, carbonate- and amine-based bases such as K_2_CO_3_, Cs_2_CO_3_, Na_2_CO_3_, DIPEA, and triethylamine led to significantly lower yields. The superior performance of KOAc can be linked to its moderate basicity,^42^ which allows the deprotonation of the terminal alkyne without causing side reactions or catalyst deactivation. Its weak coordination ability also helps stabilize the Pd and Cu species during the reaction.^43,44^ Based on these observations, KOAc was chosen as the optimal base for the catalytic experiments. The solvent effect was studied using KOAc as the base (Table S8). Among the tested solvents, DMF and DMSO showed the highest activity, with DMSO giving the highest yield, while DMF gave a lower yield of 38%. Both are polar aprotic solvents that can dissolve the substrates, allowing the catalytic cycle to proceed efficiently. In contrast, MeCN and THF showed negligible conversion, likely due to their less effective solvation of the base and charged intermediates. Mixed or less polar solvents, including DMF/H_2_O, toluene, and ethanol, resulted in poor yields. Among the tested combinations, DMSO with KOAc afforded the highest yield, confirming this pair as the ideal condition for the reaction. This is attributed to the balance between high solvent polarity and moderate base strength, which enables efficient substrate activation without deactivating the catalyst. We also investigated the influence of reactant concentration on the Sonogashira cross-coupling reaction (Figure 4c, Table S9). Increasing the concentration from 0.10 to 0.36 M resulted in a marked increase in product yield, consistent with higher effective collision frequencies and more efficient utilization of cooperative Pd–Cu active sites under kinetically controlled conditions. Beyond this concentration, further increases led to a gradual decrease in product yield, accompanied by enhanced side-product formation, indicating a transition to a selectivity-limited regime. In Sonogashira cross-coupling reactions, the predominant side product is the Cu­(I)-mediated Glaser-type homocoupling of terminal alkynes, which displays a higher apparent kinetic order with respect to alkyne concentration. As a result, substrate-rich conditions disproportionately favor homocoupling over cross-coupling. This kinetic competition, together with partial saturation of active sites and diffusion limitations within the porous COF framework, could explain the observed trade-off between activity and selectivity at higher concentrations. To better understand how the reaction evolves with time, the reaction was carried out over 8, 16, and 24 h under identical conditions and quenched at the specified time points. This approach first revealed a clear trend for the DMSO/KOAc system, which then motivated further studies involving changes in solvent and base. Under DMSO/KOAc conditions, the yield increased gradually with time, from 57% conversion at 8 h to 85% at 16 h, and reached over 95% yield at 24 h, as depicted in Figure 4d and Table S10. This steady increase indicates that the reaction is mainly limited by time and proceeds cleanly to completion, with no evidence of significant side reactions or product degradation. When the solvent was changed from DMSO to DMF while keeping KOAc as the base, the reaction became noticeably slower. Yields were much lower at early time points (21% at 8 h and 38% at 16 h), with only a small increase at 24 h (41%) and a more pronounced rise observed only after extended reaction time (61% at 36 h) (Figure 4e, Table S10). This behavior suggests that DMF provides a less favorable reaction environment, likely due to differences in solvent polarity and solvation that reduce the effective reactivity of the acetate base or stabilize key intermediates less efficiently. Importantly, the continued increase in reaction yield at longer times shows that KOAc remains sufficiently mild to preserve product stability, even though the overall reaction rate is reduced. A very different trend was observed when KOAc was replaced by the stronger base KOH in DMF. Although a moderate conversion was obtained at 8 h (42%), longer reaction times led to a clear decrease in reaction yield, dropping to 40% at 16 h and 32% at 24 h (Figure 4f, Table S10). This nonmonotonic behavior suggests that, while KOH can initially accelerate the reaction, prolonged exposure to strongly basic conditions may be detrimental. The use of KOH likely promotes competing side reactions, product degradation, or catalyst deactivation. To demonstrate the cooperativity between Pd and Cu, as control experiments, Cu@pyPPC–NaCl and Pd@pyPPC–NaCl were also evaluated under batch conditions (24 h), as shown in Table S11. In the absence of copper, with Pd@pyPPC–NaCl the reaction afforded 48.5% yield, confirming that palladium alone can promote the coupling reaction but with reduced efficiency. Cu@pyPPC–NaCl, on the other hand, showed much lower yield below 6.6% accompanied by a trace of amount of side product formation, the catalytic activity of which was attributed to the small amount of Cu­(I) species.

**Figure 4:**
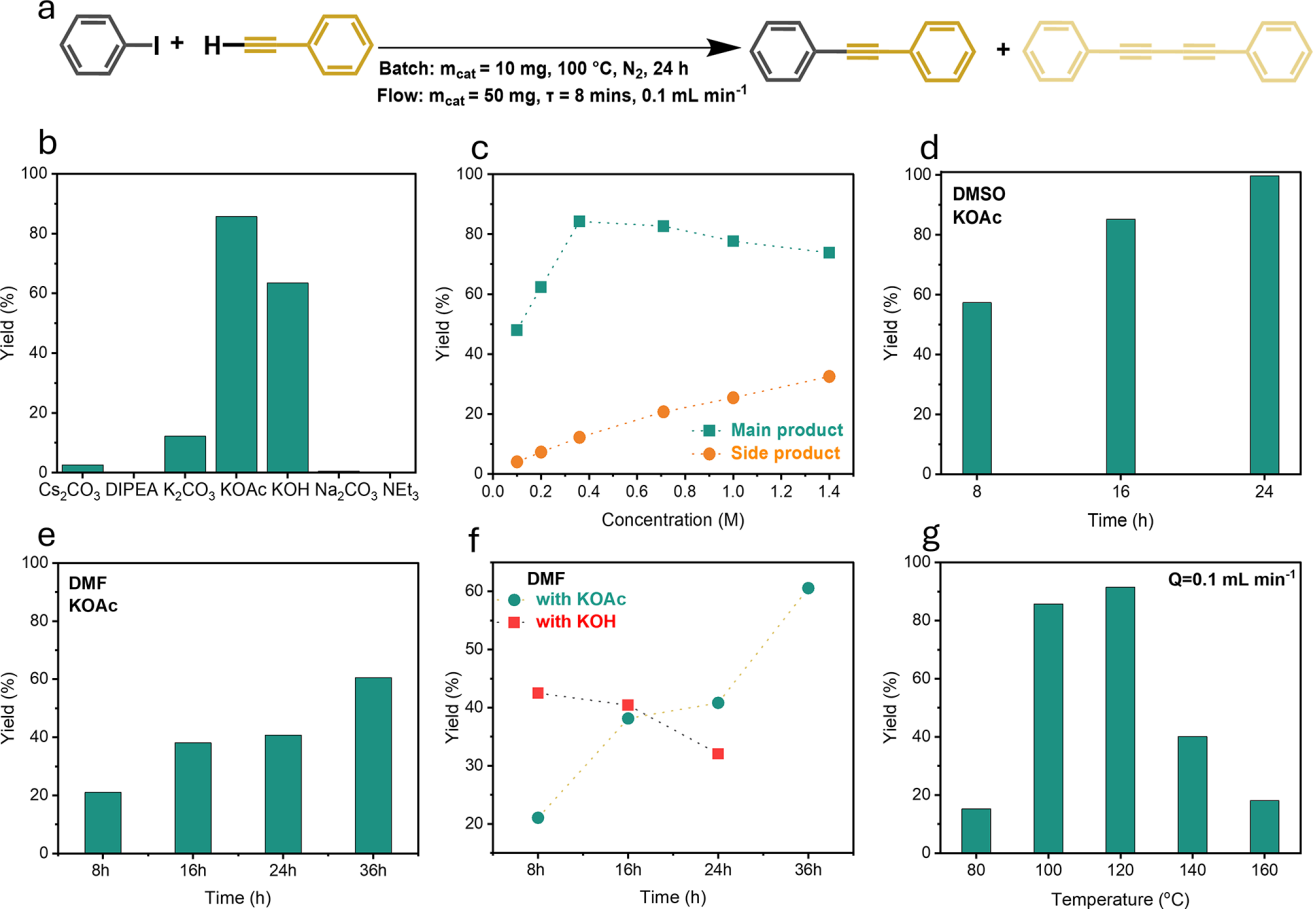
Catalytic performance of Pd-Cu@pyPPC–NaCl in the Sonogashira–Hagihara cross-coupling. (a) Reaction scheme (b) Base screening for the coupling of iodobenzene and phenylacetylene. (c) Effect of substrate concentration on the yields of the cross-coupled product and homocoupling byproduct. (d–f) Time-dependent yield profiles obtained using different base/solvent combinations: (d) KOAc in DMSO, (e) KOAc in DMF, and (f) KOH in DMF. (g) Temperature dependence under continuous-flow conditions.

This result suggests that a further increase in the proportion of Cu­(I) species through controlled reduction can lead to improved yields. The enhanced catalytic performance of Pd–Cu@pyPPC–NaCl relative to the monometallic Pd system indicates a clear Pd–Cu synergistic effect in the Sonogashira cross-coupling reaction (Figure 5d). The bimetallic catalyst exhibits significantly faster kinetics and higher yields, which can be attributed to the role of Cu in forming copper acetylide intermediates that facilitate rapid transmetalation with Pd­(II) species. This pathway bypasses the kinetically demanding direct alkyne activation at Pd centers, thereby accelerating the overall catalytic cycle. The proposed mechanism is based on experimentally supported kinetic trends and control experiments, and is consistent with established Sonogashira catalytic cycles, although direct mechanistic verification remains beyond the scope of this study. In addition, we also performed the reaction under air using Pd–Cu@pyPPC–NaCl for 24 h, resulting in 68% yield, which demonstrates that an inert atmosphere improves catalytic performance but is not strictly required for product formation.

**Figure 5:**
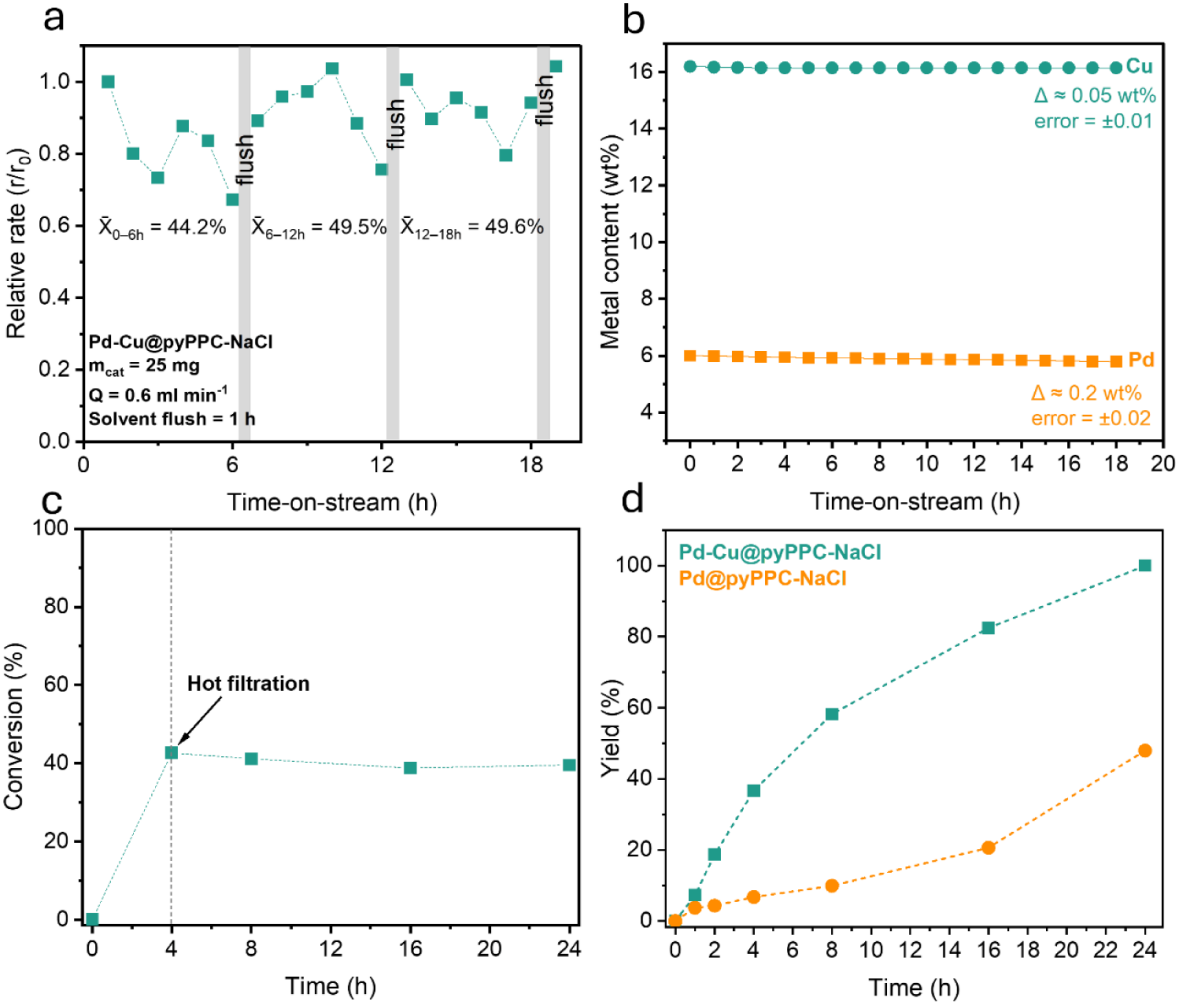
Catalyst performance and stability studies. (a) Time-on-stream relative rate profile with periodic solvent flushing. (b) Metal content of Pd and Cu during time-on-stream analysis. (c) Hot filtration test. (d) Kinetic comparison of mono- and bimetallic catalysts in Sonogashira cross-coupling.

The substrate scope of the Sonogashira cross-coupling reaction using Pd–Cu@pyPPC–NaCl was examined under the optimized reaction conditions using a set of aryl iodides with diverse functional groups (Table 1). The substrates were selected to establish structure–reactivity trends by varying electronic properties and functional-group compatibility, covering electron-withdrawing, electron-donating, coordinating, and protic substituents. Electron-poor aryl iodides reacted most efficiently, with 4-iodobenzonitrile and 4-iodobenzotrifluoride reaching near-quantitative conversion (99%). In contrast, substrates bearing strongly coordinating or acidic groups, such as 4-iodobenzoic acid, 4-iodophenol, and 4-iodoboronic acid, showed low conversions. Electron-donating substrates gave moderate to good results, with 4-iodoanisole and 4-iodotoluene reaching around 70% conversion, whereas aniline derivatives reacted poorly. The Pd–Cu@pyPPC–NaCl catalyst was also evaluated under continuous-flow conditions in a packed-bed reactor using the setup shown in Scheme S1, with the reaction temperature varied at a fixed total flow rate of 0.10 mL min^–1^, corresponding to a liquid hourly space velocity (LHSV) of 7.5 h^–1^ and a residence time of 8 min. As shown in Figure 4g and Table S10, increasing the temperature from 80 to 120 °C led to a pronounced enhancement in catalytic performance. Under optimized reaction conditions, diphenylacetylene was obtained at steady state in 91.45% yield, corresponding to a molar production rate of 1.79 mmol h^–1^ (0.319 g h^–1^) (Table S13). Normalization to the reactor volume (0.80 mL) afforded a space–time yield of 2.24 mol L^–1^ h^–1^ (399 g L^–1^ h^–1^), while catalyst-normalized productivity reached 35.85 mmol h^–1^ g_cat_^–1^ (6.39 g h^–1^ g_cat_^–1^). The improved performance at elevated temperature reflects accelerated reaction kinetics and more effective utilization of the Pd–Cu bimetallic active sites under flow conditions. Further increases in temperature beyond 120 °C resulted in a decline in yield, indicating the increasing contribution of side reactions and reduced selectivity under harsher thermal conditions. Notably, the optimal reaction temperature under continuous-flow operation is higher than that employed in batch reactions (100 °C), which can be attributed to the shorter residence time in the flow reactor, necessitating higher thermal input to achieve comparable conversion. Apparent turnover frequencies of 61.6 h^–1^ (Pd basis) and 14.1 h^–1^ (Cu basis) were calculated assuming full metal accessibility. Corresponding turnover numbers of 15.4 (Pd) and 3.53 (Cu) were obtained after 15 min on stream. Extending the analysis to prolonged operation, integration of the time-on-stream profile over 18 h gives cumulative turnover numbers of 969.7 (Pd basis) and 221.9 (Cu basis), compared to 1108.8 and 253.8 estimated from a constant-rate approximation. This difference reflects the dynamic nature of catalytic operation under continuous-flow conditions, including transient variations associated with solvent flushing and subsequent recovery phases. Accounting for these effects provides a more representative description of catalyst performance over extended operation. Importantly, the catalyst maintains a high average activity throughout the entire period, demonstrating its robustness and the sustained efficiency of the Pd–Cu active sites under continuous-flow conditions.

**Table 1:**
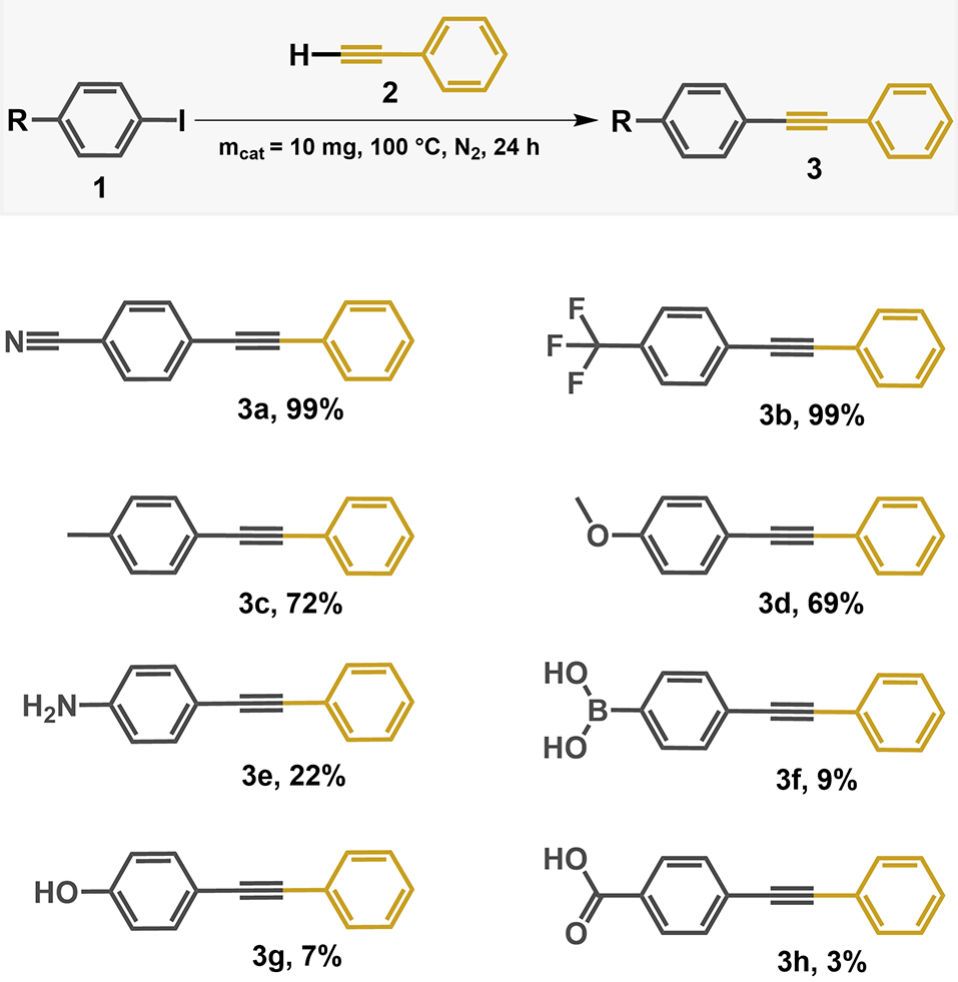
Product Yields Obtained in the Pd–Cu@pyPPC–NaCl Catalyzed Sonogashira–Hagihara Coupling, Illustrating the Substrate Scope of Accessible Diaryl Alkynes^a^

a Note: The aryl fragment originating from the terminal alkyne is highlighted in yellow, while the aryl iodide–derived fragment is shown in gray. Reaction conditions: aryl iodide (0.5 mmol, 1.0 equiv), phenylacetylene (1.05 equiv), KOAc (2.0 equiv), solvent (0.36 M), Pd–Cu@pyPPC–NaCl (10 mg), undecane as internal standard, 100 °C, 24 h, under inert atmosphere.

The stability of Pd–Cu@pyPPC–NaCl was evaluated under continuous-flow conditions (m_cat_ = 25 mg, Q = 0.6 mL min^–1^) over 18 h of time-on-stream, with periodic solvent flushing intervals (Figure 5a). The catalyst maintains a relatively stable normalized reaction rate throughout the experiment, with only moderate fluctuations observed between operating cycles. Notably, these transient decreases in activity coincide with extended reaction periods and are fully restored upon solvent flushing, indicating that the loss in activity is reversible. This behavior suggests that the temporary deactivation originates from the accumulation of strongly adsorbed intermediates or byproducts within the porous framework, leading to partial blockage of active sites or diffusion limitations. The effective regeneration of catalytic performance after each flushing step confirms that the active Pd–Cu sites remain structurally intact and accessible. Furthermore, the consistent average conversion values across each interval demonstrate the absence of long-term deactivation, which highlights the robustness of the catalyst under continuous operation. Consistent with this stability, ICP-OES analysis shows negligible leaching of Pd and Cu during time-on-stream, with only minimal variations in metal content (Figure 5b). Moreover, hot filtration experiments reveal that catalytic activity ceases upon removal of the solid catalyst, with no further increase in conversion observed (Figure 5c). These results rule out the contribution of leached species and confirm the truly heterogeneous nature of the catalytic system. In addition, post-reaction PXRD measurements after 10 consecutive catalytic cycles show no discernible changes compared to the pristine material, indicating that the crystallinity of the framework is well preserved under the reaction conditions (Figure S4). These parameters demonstrate that Pd–Cu@pyPPC–NaCl delivers high catalytic activity and stability in the Sonogashira cross-coupling reaction thanks to the synergistic activity of Pd and Cu sites embedded within the conjugated COF structure. High activity and selectivity are maintained across a broad substrate scope, and the catalyst remains stable under continuous-flow operation, which indicates suitability for conditions relevant to practical and industrial use. Although DMSO provides the highest yields in the present study, the catalytic system is well-positioned for future use with greener, industry-compatible polar aprotic solvents such as Cyrene (dihydrolevoglucosenone), as a more sustainable alternative without compromising process requirements. These features position this bimetallic COF platform as a robust and adaptable heterogeneous catalyst for industrial cross-coupling applications.

## Conclusions

We report a new strategy for the synthesis of bimetallic phthalocyanine-based covalent organic frameworks in which the coordination environment and spatial arrangement of metal ions are precisely controlled. This design enables the stable incorporation of distinct cavity-bound and pore-bound metal species while preventing the formation of aggregated metal domains. The cooperative catalytic behavior arising from this structural arrangement enabled high catalytic activity in Sonogashira–Hagihara cross-coupling reaction under both batch and continuous-flow conditions. More broadly, this work demonstrates that the combination of mixed-metal ionothermal COF synthesis with controlled secondary metalation provides a general platform for constructing structurally defined, bimetallic frameworks with high catalytic activity, thus effectively expanding the design space of COF-based heterogeneous single-atom catalysts.

## Experimental Section

### Materials

All chemicals were purchased from Sigma-Aldrich and used as received without further purification. Zinc­(II) chloride (puriss., crystalline powder, 98–100.5%) and anhydrous metal chloride (CuCl_2_; >99.99%, trace-metals basis) were employed as metal precursors for COF synthesis. 1,2,4,5-Tetracyanopyrazine was synthesized following a previously reported procedure.^1^ Sodium chloride (ACS reagent grade, ≥99%) and all organic solvents (DMF, DMSO, ethanol, acetone, acetonitrile) were obtained from commercial suppliers and used without further purification. Standard aqueous solution of PdCl_2_ (1000 ppm concentration) was used for postsynthetic metal impregnation and for generating ICP-OES calibration curves.

### Characterization Techniques

#### Powder X-ray Diffraction (PXRD)

Powder X-ray diffraction (PXRD) patterns were acquired on a STADIP microarea X-ray diffractometer equipped with Cu Kα radiation (λ = 0.154 nm). Data were collected over a 2θ range of 2–60° (or up to 70° when required) using a step size of 1.0° and a counting time of 30 s per step.

#### Fourier-Transform Infrared Spectroscopy (FT-IR)

FT-IR spectra were measured on a PerkinElmer Spectrum Two spectrometer using ATR mode to confirm framework formation and phthalocyanine macrocycle signatures.

#### Thermogravimetric Analysis (TGA)

TGA was carried out on a Mettler Toledo TGA/DSC 3+ instrument from 25 to 1000 °C at a heating rate of 10 °C min^–1^ under flowing air or nitrogen (50 mL min^–1^).

#### BET Surface Area and Porosity

The textural properties of the samples were evaluated using N_2_ adsorption–desorption isotherms recorded at 77 K on a Micromeritics 3Flex Surface Characterization Analyzer. Prior to analysis, all materials were activated under vacuum at 120 °C for 16 h. Specific surface areas were determined using BET and Langmuir models by selecting the pressure region that meets the Rouquerol criteria, where V­(1 – P/P_0_) increases steadily with P/P_0_. Pore-size distributions were obtained from the N_2_ isotherms using the NLDFT method (Carbon, 2D-NLDFT heterogeneous surface model) implemented in the Saieus software.

#### Scanning Electron Microscopy (SEM) and EDX Mapping

The morphology of the materials was investigated by scanning electron microscopy (SEM, Thermo Fisher Scientific FEI XL30 SFEG), and elemental composition and spatial distribution were determined using energy-dispersive X-ray spectroscopy (EDX, Oxford Instruments AZtec Advanced equipped with an X-MAX silicon drift detector with an active area of 150 mm^2^ and an energy resolution of 127 eV).

#### Inductively Coupled Plasma Optical Emission Spectroscopy (ICP-OES)

ICP-OES measurements were performed on a PerkinElmer Optima 7000 DV system. Metal loadings of all three bimetallic COFs were determined as detailed below.

#### X-ray Photoelectron Spectroscopy (XPS)

XPS measurements were carried out on an Axis Supra (Kratos Analytical) using the monochromated Kα X-ray line of an Aluminum anode. The pass energy was set to 40 eV with a step size of 0.15 eV. The samples were electrically insulated and charge neutralization—using a low energy electron flood—gun was used, followed by adequate energy referencing.

#### X-ray Absorption Spectroscopy (XAS)

The X-ray absorption measurements were performed in transmission mode at the Cu K-edge (8979 eV) at the SuperXAS beamline of the Swiss Light Source (Villigen, Switzerland) using 15 cm long ionization chambers filled with 1 bar N2 and 1 bar Ar. The polychromatic beam resulting from the 2.1 T magnet was collimated using a Pt-coated collimating mirror, subsequently monochromatized by a channel-cut Si(111) crystal of the quick scanning monochromator and finally focused using a Pt-coated toroidal mirror to a spot size of 1000 μm × 150 μm. Cu foil, mounted between the second and the third ionization chambers was used for internal energy calibration. Spectra were collected at 1 Hz for 300 s and averaged to improve data quality. Basic data reduction (i.e., encoder analysis, calibration, normalization, interpolation) was performed with the ProQEXAFS software.^2^

#### Gas Chromatography–Mass Spectrometry (GC–MS)

Catalytic evaluations were carried out in both batch and continuous-flow reactors, and the reaction products were analyzed by Gas Chromatography–Mass Spectrometry (GC–MS) using a PerkinElmer Clarus 560 gas chromatograph coupled to a Clarus 560 S mass spectrometer.

### Synthetic Methods

Cu@pyPPC–NaCl was synthesized using a general mixed-metal ionothermal procedure, in which 50 mg (0.278 mmol) of 1,2,4,5-tetracyanopyrazine was mixed with 0.14 mmol of the corresponding anhydrous metal chloride precursor CuCl_2_ (18.8 mg) together with 1225 mg (8.99 mmol) of ZnCl_2_ and 246 mg (4.21 mmol) of NaCl. The mixture was ground thoroughly and transferred into a Pyrex ampule (3 × 12 cm) under an inert atmosphere. The ampule was subjected to three vacuum/argon refill cycles, evacuated, and flame-sealed. The sealed tube was placed in a box furnace, heated to 300 °C at a rate of 3 °C min^–1^ and held for 5 h, then heated to 400 °C (3 °C min^–1^) and maintained at this temperature for 24 h to form Cu@pyPPC–NaCl. After slow cooling to room temperature, the ampule was opened carefully, and the resulting black solid was collected, ground into a fine powder, and washed thoroughly with deionized water to remove ZnCl_2_ and NaCl. The material was then stirred in 200 mL of deionized water for 24 h, filtered, and washed sequentially with 500 mL of water followed by 200 mL each of acetone and ethanol. All final Cu@pyPPC–NaCl powders were dried under vacuum at 90 °C for 24 h.

### Postsynthetic Metal Impregnation

Postsynthetic metal loading was carried out using aqueous standard metal solutions. In a typical procedure, 50 mg of the activated Cu@pyPPC–NaCl material was dispersed in 5 mL of a metal salt solution prepared from commercial 1000 ppm stock standards of Pd. The suspension was stirred at room temperature for 24 h to allow metal uptake into the COF pores. After impregnation, the solid was separated by filtration and washed repeatedly with deionized water and ethanol to remove unbound metal ions. The impregnated powder was then dried under vacuum at 90 °C for 24 h. Metal uptake was quantified by ICP-OES analysis of the supernatant solution before and after impregnation, and the amount of metal incorporated into the framework was calculated by mass balance using the initial solution concentration as a reference.

### ICP-OES Sample Preparation and Metal Content Determination

For ICP-OES measurements, the Cu@pyPPC–NaCl samples were first subjected to complete thermal decomposition by heating to 1000 °C under air to remove the organic framework. The remaining residues were digested in 2.5 mL of freshly prepared aqua regia (HNO_3_:HCl = 1:3, v/v) and left to stand for 10 days to ensure complete dissolution. For analysis, 0.3 mL of the digested solution was diluted with 9.7 mL of double-deionized (DDI) water, and a blank was prepared by diluting 0.3 mL of aqua regia in the same way. Calibration standards for Cu were prepared by serial dilution of 1000 ppm stock solutions to 200, 120, 50, 20, 5, and 0.5 ppm. The measured concentrations were used to calculate the metal contents of the original COF samples. For samples subjected to postsynthetic metal impregnation, the secondary metal loading (Pd) was determined from the uptake during impregnation. The concentration of metal remaining in the supernatant was measured by ICP-OES, and the incorporated amount was obtained by mass balance relative to the initial solution. Calibration curves were prepared from 1000 ppm aqueous standards, and the final loading values were corrected for blanks and dilution.

### Structural Analysis

Structural analysis of Cu@pyPPC–NaCl and Pd–Cu@pyPPC–NaCl was completed with the use of Materials Studio Software. The periodic two-dimensional structure of Cu–pyPPC–NaCl was modeled in the tetragonal *P4/mmm* space group. The structure was geometrically optimized with the use of universal force field, rendering unit cell parameters of a = 10.48 Å and c = 3.42 Å. Based on this optimized model, the structure of Pd–Cu@pyPPC–NaCl was simulated by incorporating palladium atoms in the pores, coordinated to nitrogen atoms of the framework, and with chlorine atoms completing the planar-square coordination environment. Due to the presence of multiple symmetry equivalent positions in the pores, partial occupancies were assigned to Pd and Cl atoms, with values consistent with the ones obtained from the ICP-OES analysis.

### Catalytic Test Procedure for the Sonogashira Coupling Reaction

The Sonogashira coupling reaction was performed in a standard round-bottom flask equipped with a magnetic stir bar. DMSO (1.4 mL) was added first, followed by the catalyst (10 mg of Pd–Cu@pyPPC–NaCl) and KOAc (1.0 mmol, 96.21 mg). Phenylacetylene (0.525 mmol, 52.56 mg) was added next, and iodobenzene (0.50 mmol, 100 mg) was added last. This order of addition ensured proper catalyst dispersion, effective base–alkyne interaction, and controlled introduction of the aryl halide. The reaction mixture was briefly degassed and maintained under an inert atmosphere, then heated at 100 °C with stirring for 24 h. After cooling to room temperature, the mixture was diluted with water and extracted with dichloromethane. The organic phase was dried over Na_2_SO_4_, filtered, and analyzed by GC–MS to determine conversion, yield, and product selectivity.

### Continuous-Flow Procedure for the Sonogashira Coupling Reaction

The continuous-flow Sonogashira coupling reactions were carried out using a packed-bed reactor (Scheme S1). The reactor (internal volume = 0.8 mL) was packed with 50 mg of Pd–Cu@pyPPC–NaCl between two layers of plates made of filter paper. A single HPLC pump equipped with a dual-inlet head was used to deliver the feed solutions. The organic stream, consisting of iodobenzene (1.0 mmol) and phenylacetylene (1.05 mmol) dissolved in DMSO (8 mL), and the aqueous stream containing KOAc (2.0 mmol) in water (1 mL), were introduced through inlets A and B, respectively. A small amount of water was intentionally included to improve KOAc solubility and to minimize the risk of salt accumulation and reactor clogging, while keeping the total reaction volume equivalent to the batch conditions. The pump was set to an 8:1 volumetric mixing ratio (A:B) to reproduce the reaction composition, and the combined stream was delivered to the packed-bed reactor at a total flow rate of 0.10 mL min^–1^. The reactor temperature was maintained at 120 °C. These conditions were selected based on preliminary optimization experiments (Figures S5–S12). After reaching steady-state operation, the reactor effluent was collected over defined time intervals and analyzed by GC–MS to determine conversion, yield, and product distribution.

## Supplementary Material



## Data Availability

The data supporting the findings of this study are openly available in Zenodo at https://doi.org/10.5281/zenodo.20564637

## Notes

Additional experimental procedures, figures, and referenced material (PDF)
