# Supplementary material for: Pd–Cu Single-Atom Catalysts in Covalent Organic Frameworks for Sonogashira Cross-Coupling Reaction
Source: Chem Mater. 2026 Jun 24;38(13):6434–45. doi: 10.1021/acs.chemmater.6c00391 (PMC13374044; doi:10.1021/acs.chemmater.6c00391)
Supplement: Supplementary file 1 [file cm6c00391_si_001.pdf]

Pd–Cu Single-Atom Catalysts in Covalent Organic Frameworks for Sonogashira Cross-Coupling Reaction

Murad Najafov,[1,2] Daniele Bonavia,[3] Felipe Gándara,[4] Maarten Nachtegaal,[3,5,6] Mounir Mensi[7] and Ali Coskun[1,2]*

[1]Department of Chemistry, University of Fribourg, Chemin du Musee 9, Fribourg 1700, Switzerland

[2]National Centre of Competence in Research (NCCR) Catalysis, University of Fribourg, Fribourg 1700, Switzerland

[3]Center for Photon Science, PSI, Villigen, CH-5232, Switzerland

[4]Materials Science Institute of Madrid—CSIC, Sor Juana Inés de la Cruz 3, Madrid 28049, Spain

[5]Center for Energy and Environmental Sciences, PSI, Villigen, CH-5232, Switzerland,

[6]Department of Chemistry, ETH Zürich, CH-8093, Zürich, Switzerland

[7]Institute of Chemical Sciences and Engineering (ISIC), École Polytechnique Fédérale de Lausanne, Sion 1950, Switzerland

*Correspondence: ali.coskun@unifr.ch.

SUPPORTING INFORMATION

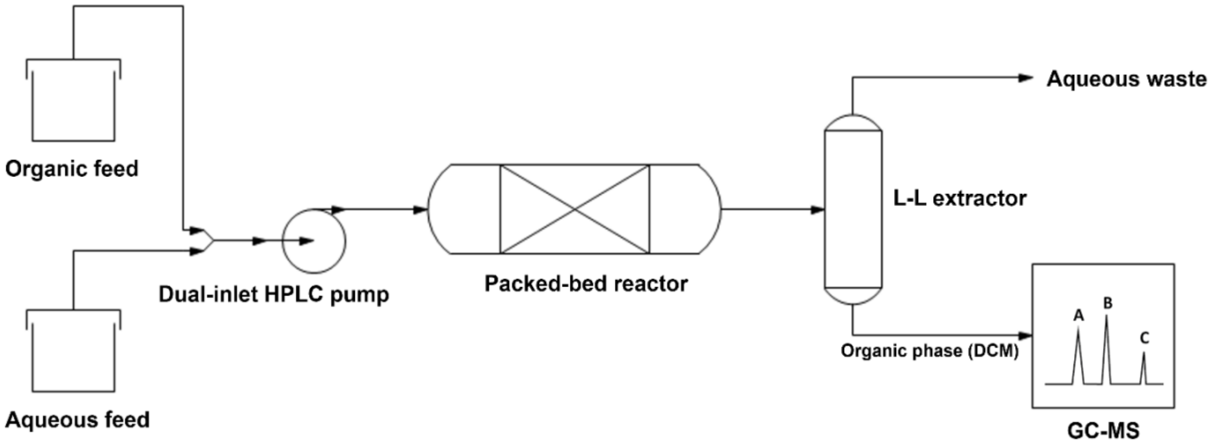

Scheme S1. Process Flow Diagram (PFD) of the continuous-flow setup used for the Sonogashira coupling reaction.

Table S1. Atomic Coordinates and occupancies

Compound: Pd-Cu@pyPPC-NaCl

Crystal system: Tetragonal

Space group (H–M): *P4/mmm*

Space group number: 123

a = 10.482 Å, b = 10.482 Å, c = 3.422 Å

α = 90°, β = 90°, γ = 90°

Atom label	Element	x	y	z	Site Occupancy	Site multiplicity
Cu1	Cu	0.00000	0.00000	0.00000	1.00	1
N2	N	0.77329	0.22671	0.00000	1.00	4
N3	N	0.00000	0.18967	0.00000	1.00	4
C4	C	0.10405	0.26618	0.00000	1.00	8
C5	C	0.06592	0.39092	0.00000	1.00	8
N6	N	0.13615	0.50000	0.00000	1.00	4
Pd7	Pd	0.31441	0.59259	0.00000	0.0625	8
Cl8	Cl	0.50000	0.64515	0.00000	0.125	4
Cl9	Cl	0.40843	0.59157	0.00000	0.125	4

Table S2. Atomic bond lengths and types.

Atom 1	Atom 2	Bond length (Å)	Symmetry	Bond type
Cu1	N3	1.988	—	S
Cu1	N3	1.988	2	S
Cu1	N3	1.988	3	S
Cu1	N3	1.988	4	S
N2	C4	1.351	3_655	A
N2	C4	1.351	5_655	A
N3	C4	1.354	—	A
N3	C4	1.354	5	A
C4	C5	1.367	—	A
C5	N6	1.360	—	A
C5	C5	1.382	5	S
N6	C5	1.360	6_565	A
N2	Pd7	2.105	2_664	S
N2	Pd7	2.105	7_556	S
N6	Pd7	2.106	1_554	S
N6	Pd7	2.106	6_566	S

Table S3. Textural parameters and metal loadings of Cu@pyPPC-NaCl before and after Pd incorporation.

**BET[a] (m^2 g^{-1})**	**S_{micro}[b] (m^2 g^{-1})**	**S_{ext} (m^2 g^{-1})**	**V_{total}[c] (cm^3 g^{-1})**	**V_{micro}[d] (cm^3 g^{-1})**	**$V_{external}$[e] (cm^3 g^{-1})**	**Cu[f] (before) (wt%)**	**Pd[f] (wt%)**	**Cu[f] (after) (wt%)**
480	385.6	93.7	0.26	0.16	0.096	16.5	6.2	16.2

[a] Brunauer–Emmett–Teller (BET) surface area calculated over the pressure range (P/P_0) of 0.01–0.11. [b] Micropore surface area calculated using the t-plot method. [c] Total pore volume obtained at P/P_0 = 0.99. [d] Micropore volume calculated using the *t*-plot method. [e] $V_{external} = V_{total} - V_{micro}$. [f] Metal contents determined by inductively coupled plasma–optical emission spectrometry (ICP-OES).

Table S4. Elemental analysis (EA) result of Pd-Cu@pyPPC-NaCl framework.

**Element**	**Computed value[a]**	**Experimental value[b]**
Carbon	44.92	35.45
Nitrogen	39.29	34.74
Hydrogen	0.94	1.65
Total	91	71.83

[a] Computed values based on the pristine pure unit cell and an average of 1 Cu atom per unit: Cu@pyPPC-NaCl ($C_{16}H_4N_{12}Cu$), [b] Measured by EA.

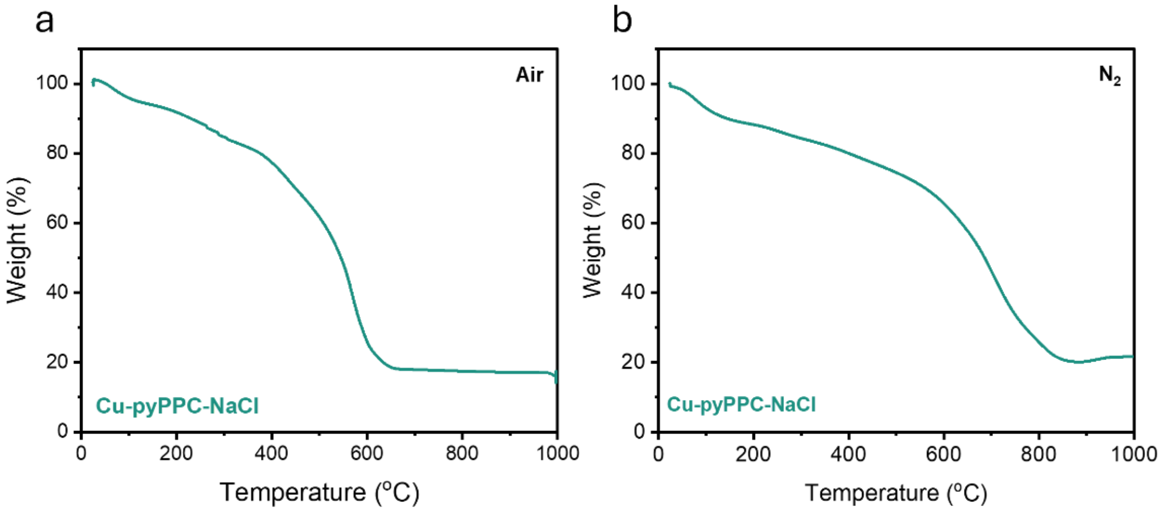

Figure S1. TGA analysis of Cu@pyPPC-NaCl in (a) air and (b) nitrogen.

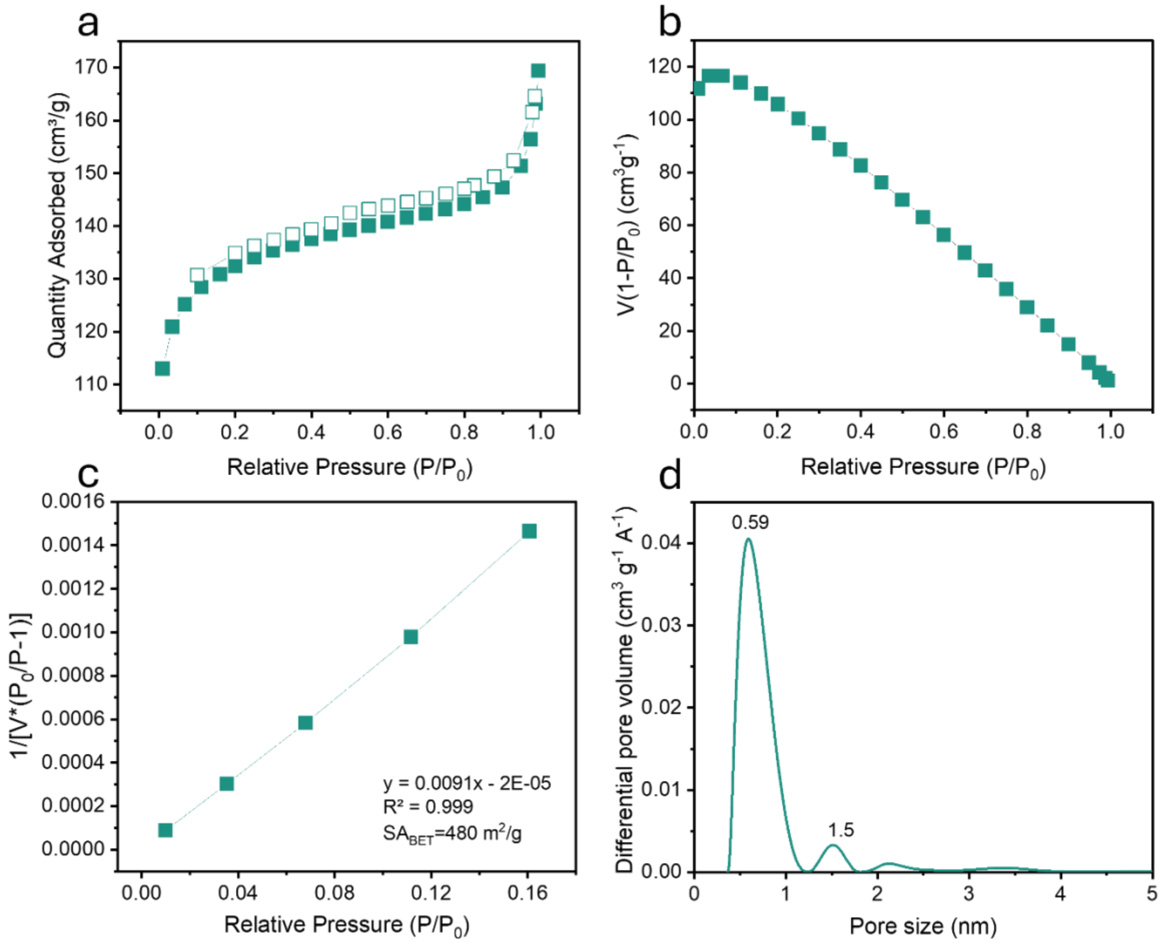

Figure S2. Textural characterization of Cu@pyPPC–NaCl derived from N_2 physisorption measurements at 77 K: (a) N_2 adsorption–desorption isotherm, (b) Rouquerol plot used to define the appropriate relative pressure range for BET analysis, (c) corresponding linear BET plot, and (d) pore size distribution calculated from the adsorption branch.

EXAFS Analysis

Analysis of the Extended X-ray Absorption Fine Structure (EXAFS) was carried out using the Larch[3] Python package. The FEFF8 code[4] was used to obtain the theoretical scattering paths from the optimized. COF structure using Cu and Pd as absorbers, which were used to fit the different contributions over the two absorption edges.

Metal foils were fitted to bulk Pd and Cu structures to obtain the passive electron amplitude reduction factor (S_0^2) for the two absorbers. Following this, first shell EXAFS fits were performed on the two edges.

For Cu, 4 N atoms form the first shell according to the optimized COF structure.

For Pd, the first and second coordination shells are formed by, respectively, 2 N (R =2.11 Å) and 2 Cl (R =2.34 Å) atoms. Separating the contributions for these two shells has proven complicated because of the small difference between the atom-absorber distances and the relatively light atomic weight of the atoms involved. The approach adopted to circumvent this problem was treating the two shells as one, with an expected overall coordination of 4. All fit results are provided in Table S5.

Table S5. EXAFS fit parameters and uncertainties for the first shell fits over Cu K-edge.

Edge	Shell	ΔE (eV)	S_0^2	CN	ΔR (Å)	σ^2 ($Å^{-2}$)
**Cu-K**	1st	2 ± 3	0.98	3.8 ± 0.8	-0.03 ± 0.01	0.004 ± 0.002

For Pd, the first and second coordination shells are formed by, respectively, 2 N (R =2.11 Å) and 2 Cl (R =2.34 Å) atoms, correponding fitting approach was performed using the this coordination number and Debye-Waller for both the first and second single scattering paths (2 N, 2 Cl). In addition, the variation in distance from the bulk structure ΔR was considered correlated, applying an isotropic expansion of the type ΔR = Reff*α to both scattering paths. The fit results are reported in Table S6.

Table S6. EXAFS fit parameters and uncertainties for the first and second shell fits over Pd K-edge.

Edge	Shell	ΔE (eV)	S_0^2	CN	α	σ^2 (Å^{-2})
**Pd-K**	1st	3.4 ± 1.8	0.77	2.7 ± 0.4	-0.062 ± 0.005	0.011 ± 0.001
	2nd					

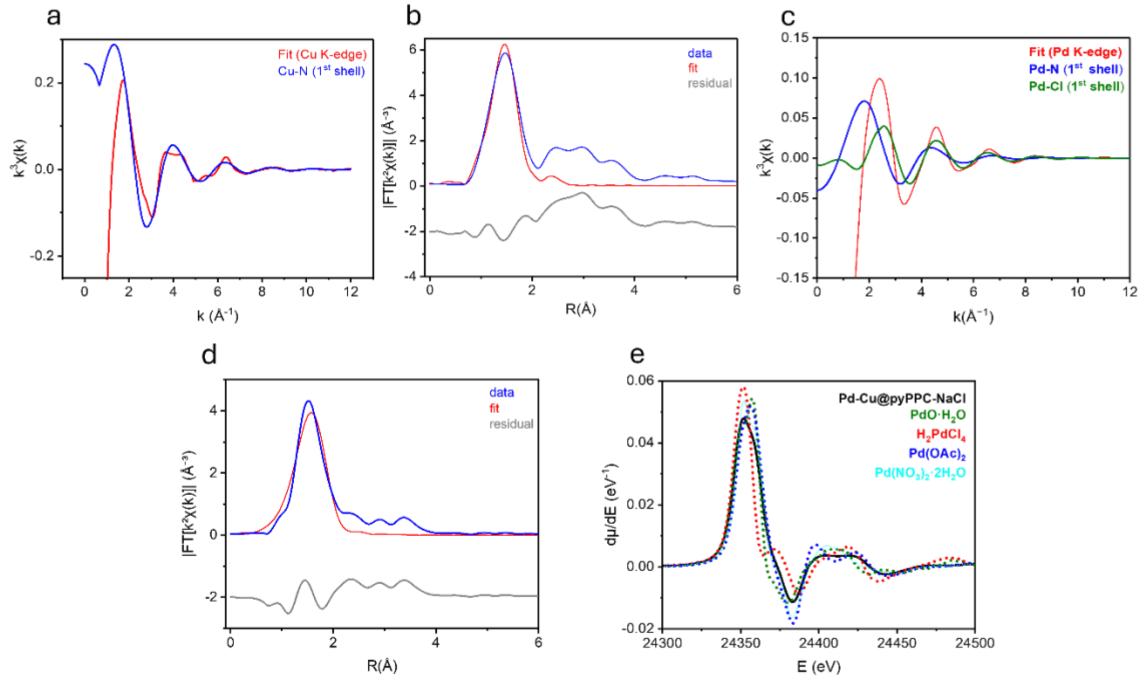

Figure S3. EXAFS and XANES analysis of Cu and Pd sites. (a) Cu K-edge χ(k) EXAFS fit. (b) |FT[χ(k)]| of the Cu K-edge EXAFS with fit and residual. (c) Pd K-edge χ(k) EXAFS fit. (d) |FT[χ(k)]| of the Pd K-edge EXAFS with fit and residual. (e) First derivative of the Pd K-edge XANES spectra.

Table S7. Effect of base on the yield of the model reaction. Reaction conditions: Pd-Cu@pyPPC-NaCl (10 mg), solvent DMSO, inert atmosphere, T = 100 °C, reaction time = 16 h. Yields were determined by GC-MS.

Entry	Base	Yield (%)
**1**	Cs_2CO_3	2.6
**2**	DIPEA	0.1
**3**	K_2CO_3	12.2
**4**	KOAc	85.7
**5**	KOH	63.5
**6**	Na_2CO_3	0.4
**7**	NEt_3	0

Table S8. Effect of solvent on the yield of the model reaction. Reaction conditions: Pd-Cu@pyPPC-NaCl (10 mg), base KOAc, inert atmosphere, T = 100 °C, reaction time = 16 h. Yields were determined by GC-MS.

Entry	Solvent	Yield (%)
**1**	DMF	11.5
**2**	DMSO	85.1
**3**	Ethanol	0.4
**4**	H_2O	0.5
**5**	MeCN	0.6
**6**	THF	0
**7**	Toluene	0.4

Table S9. Effect of reactant concentration on the yields of the main product and side product for the model reaction. Reaction conditions: Pd-Cu@pyPPC-NaCl (10 mg), base KOAc, solvent DMSO, inert atmosphere, T = 100 °C, reaction time = 16 h. Yields were determined by GC-MS.

Entry	Concentration (M)	Yield (%)	Side Product Yielf (%)
**1**	0.1	48.0	4.0
**2**	0.2	62.4	7.3
**3**	0.36	84.2	12.2
**4**	0.71	82.7	16.7
**5**	1	77.7	21.4
**6**	1.4	73.8	25.5

Table S10. Effect of reaction time on the yield of the model reaction for different solvent–base pairs. Reactions were conducted using Pd–Cu@pyPPC-NaCl (10 mg) under inert atmosphere at 100 °C for the indicated reaction times. Each data point corresponds to an independent experiment. Yields were determined by GC–MS.

Entry	Time (h)	Solvent	Base	Yield (%)
**1**	8	DMSO	KOAc	57.3
**2**	16	DMSO	KOAc	85.2
**3**	24	DMSO	KOAc	99.7
**4**	8	DMF	KOH	42.5
**5**	16	DMF	KOH	40.4
**6**	24	DMF	KOH	32.0
**7**	8	DMF	KOAc	21.0
**8**	16	DMF	KOAc	38.2
**9**	24	DMF	KOAc	40.8
**10**	36	DMF	KOAc	60.6

Table S11. Control experiments evaluating the effect of catalyst composition and reaction atmosphere on the yield of the model reaction. Reactions were performed using Pd@pyPPC-NaCl (10 mg) or Cu@pyPPC-NaCl (10 mg) under inert atmosphere, while the bimetallic Pd–Cu@pyPPC-NaCl (10 mg) catalyst was evaluated under air. All reactions were conducted with KOAc as the base in DMSO at 100 °C for 24 h. Yields were determined by GC–MS.

Entry	Catalyst	Atmosphere	Yield (%)
**1**	Pd@pyPPC-NaCl	Inert	48.5
**2**	Cu@pyPPC-NaCl	Inert	6.6
**3**	Pd-Cu@pyPPC-NaCl	Air	68.8

Table S12. Effect of reaction temperature on the yield of the model reaction under continuous-flow conditions. Reactions were performed in a packed-bed reactor using Cu–Pd@pyPPC–NaCl (50 mg) with KOAc as the base, feed kept under inert atmosphere. The total flow rate was 0.10 mL min^{-1}, corresponding to a residence time of 8 min. Yields were determined by GC–MS.

Entry	Condition	Yield (%)
**1**	80	15.3
**2**	100	85.7
**3**	120	91.5
**4**	140	40.1
**5**	160	18.1

Operating parameters and flow conditions

Continuous-flow experiments were conducted at a total volumetric flow rate of 0.10 mL min^{-1} (6.0 mL h^{-1}). The organic and aqueous feed streams were introduced at a fixed volumetric ratio of 8:1, corresponding to flow rates of 0.0889 mL min^{-1} (5.33 mL h^{-1}) for the organic stream and 0.0111 mL min^{-1} (0.667 mL h^{-1}) for the aqueous stream. The packed-bed reactor had an effective internal volume of 0.80 mL (8.0×10^{-4} L), which corresponds to a residence time of 8 min at the applied flow rate. For analytical quantification, reactor effluent was collected over a 15 min steady-state interval, yielding a total collected volume of 1.5 mL.

Total flow rate: Q_{Total} = 0.1mL min^{-1} = 6 mL h^{-1} = 0.006 L h^{-1}

Feed ratio: Organic feed / Aqueous feed = 8 / 1

Sample collection time: t = 15 min = 0.25 h

Reactor volume: V_R = 0.8 mL = 8×10^{-4} L

Mass of catalyst: m_{cat} = 50 mg = 0.050 g

$$\frac{Q_{Organic}}{Q_{Total}} = \frac{8}{9}$$

$$\frac{Q_{Aqueous}}{Q_{Total}} = \frac{1}{9}$$

$$Q_{Organic} = 0.1 * \frac{8}{9} = 0.0888889 \text{ mL min}^{-1} = 5.33333 \text{ mL h}^{-1} = 0.00533333 \text{ L h}^{-1}$$

$$Q_{Aqueous} = 0.1 * \frac{1}{9} = 0.0111111 \text{ mL min}^{-1} = 0.666667 \text{ mL h}^{-1} = 0.000666667 \text{ L h}^{-1}$$

Total volume of product collected in 15 mins:

$$V_{collected} = 0.1 * 15 = 1.5$$

Molar flow rates and material balance

Molar flow rates of iodobenzene and product were determined from GC–MS analysis using an internal standard. Based on the measured product amount collected over the 15 min interval, the steady-state molar flow rate of diphenylacetylene was calculated as 1.79242 mmol h^{-1}. The corresponding inlet molar flow rate of iodobenzene was 1.9600 mmol h^{-1}, while the outlet molar flow rate of unreacted iodobenzene was 0.16758 mmol h^{-1}, consistent with near-quantitative conversion. All molar flow rates were calculated assuming steady-state operation during the sampling interval.

Table S13. Steady-state molar flow rates of reactant and product used for reactor mass-balance and performance evaluation under continuous-flow conditions.

Stream	Molar flow rate (mol/min)	Molar flow rate (mol/h)	Molar flow rate (mmol/h)
Iodobenzene inlet	3.2667×10^{-5}	1.9600×10^{-3}	1.9600
Iodobenzene outlet	2.7930×10^{-6}	1.6758×10^{-4}	0.16758
Product outlet	2.9874×10^{-5}	1.79242×10^{-3}	1.79242

Residence time and liquid hourly space velocity

The liquid hourly space velocity (LHSV) was calculated as the ratio of the total volumetric flow rate to the reactor volume. At a total flow rate of 6.0 mL h^{-1} and a reactor volume of 0.80 mL, the LHSV was 7.5 h^{-1}. The corresponding residence time, defined as the inverse of LHSV, was therefore 0.133 h (8 min), in agreement with the residence time derived independently from volumetric considerations. This confirms the internal consistency of the flow reactor parameters.

Given τ = 8 min and V_R = 0.8 mL = 8 * 10^{-4} L

Liquid hourly space velocity (LHSV):

$$\mathrm{LHSV} = \frac{\mathrm{Q}}{\mathrm{V_R}} = \frac{0.00600}{8 * 10^{-4}} = 7.5\ \mathrm{h}^{-1}$$

And $\frac{1}{\mathrm{LHSV}} = 0.1333\ \mathrm{h} = 8\ \mathrm{min}$

Space-time yield and productivity metrics

The space–time yield (STY) was calculated by normalizing the steady-state molar product formation rate to the reactor volume. Using a product formation rate of 0.00179242 mol h^{-1} and a reactor volume of 8.0×10^{-4} L, a molar STY of 2.24 mol L^{-1} h^{-1} was obtained. Based on the molar mass of diphenylacetylene (178.23 g mol^{-1}), this corresponds to a mass-based STY of 399 g L^{-1} h^{-1}. Catalyst-normalized productivity was calculated by dividing the product formation rate by the catalyst mass (50 mg), yielding 35.85 mmol h^{-1} g_{cat}^{-1}, or equivalently 6.39 g h^{-1} g_{cat}^{-1} on a mass basis.

Space-time yield (STY):

$$\mathrm{STY} = \frac{\dot{n}_P}{V_R} = \frac{0.00179242}{8 \times 10^{-4}} = 2.24 \ \ \mathrm{mol\,L^{-1}h^{-1}}$$

Mass-based STY:

$$\mathrm{STY_m} = \mathrm{STY} \times \mathrm{MW} = 2.240525 \times 178.23 = 399.33\ \mathrm{g\,L^{-1}h^{-1}}$$

Over the 15 min collection:

$$n_P(15\ \mathrm{min}) = \dot{n}_P \times 0.25\ \mathrm{h} = 1.79242 \times 0.25 = 0.448\ \mathrm{mmol}$$

Catalyst-normalized productivity:

With $m_{cat} = 0.05$ g

$$\frac{\dot{n}_P}{m_{cat}} = \frac{0.00179242\ \mathrm{mol\,h^{-1}}}{0.05\ \mathrm{g}} = 0.0358484\ \mathrm{mol\,g^{-1}h^{-1}}$$

For 15 min run

$$\frac{\dot{n}_P}{m_{cat}} = \frac{0.448105\ \mathrm{mmol}}{0.05\ \mathrm{g}} = 8.9621\ \mathrm{mmol\,g^{-1}}$$

Mass productivity:

$$\dot{m}_P = \dot{n}_P \times \mathrm{MW} = 0.00179242 \times 178.23 = 0.31946\ \mathrm{g\,h^{-1}}$$

$$\frac{\dot{m}_P}{m_{cat}} = \frac{0.31946}{0.05} = 6.3893\ \mathrm{g\,h^{-1}g_{cat}^{-1}}$$

Metal loading, TON, and TOF calculations

The packed-bed reactor contained 50 mg of catalyst with metal loadings of 6.2 wt% Pd and 16.2 wt% Cu. Based on these values, the total amounts of Pd and Cu present in the reactor were calculated as 2.91×10^{-5} mol and 1.27×10^{-4} mol, respectively. For the purpose of TON and TOF calculations, all metal atoms present in the catalyst were assumed to be catalytically accessible and active sites; therefore, the reported values represent apparent TON and TOF. Turnover frequencies (TOF) were calculated by normalizing the steady-state product formation rate (0.00179242 mol h^{-1}) to the total number of metal atoms. On this basis, TOF values of 61.6 h^{-1} and 14.1 h^{-1} were obtained for Pd and Cu, respectively. Turnover numbers (TON) over the 15 min collection interval were calculated as 15.4 (Pd basis) and 3.53 (Cu basis). TON values projected over 24 h were calculated assuming constant steady-state activity, yielding TON values of 1.48×10^3 (Pd) and 3.39×10^2 (Cu).

Catalyst mass in reactor: $m_{cat} = 50 \text{ mg} = 0.050 \text{ g}$

Loadings (wt%):

Cu = 16.2 wt%

Pd = 6.2 wt%

All metal atoms present in the catalyst were assumed to be catalytically active sites for the purpose of TON and TOF calculations.

Product formation rate:

$$\dot{n}_P = 1.79242 \text{ mmol h}^{-1} = 0.00179242 \text{ mol h}^{-1}$$

Atomic weights:

Pd = 106.42 g/mol

Cu = 63.546 g/mol

Moles of metal in the packed bed:

Pd in bed:

$$m_{Pd} = 0.050 \times 0.062 = 0.00310 \text{ g}$$

$$n_{Pd} = \frac{0.00310}{106.42} = 2.91 \times 10^{-5} \text{ mol}$$

Cu in bed:

$$m_{Cu} = 0.050 \times 0.162 = 0.00810 \text{ g}$$

$$n_{Cu} = \frac{0.00810}{63.546} = 1.27 \times 10^{-4} \text{ mol}$$

TOF based on Pd:

$$\mathrm{TOF_{Pd}} = \frac{0.00179242}{2.91 \times 10^{-5}} = 61.6\ \mathrm{h^{-1}}$$

TOF based on Cu:

$$\mathrm{TOF_{Cu}} = \frac{0.00179242}{1.27 \times 10^{-4}} = 14.1\ \mathrm{h^{-1}}$$

$\mathbf{TON_{Pd}}$ (15 min):

$$\mathrm{TON_{Pd,15min}} = \frac{4.48105 \times 10^{-4}}{2.91 \times 10^{-5}} = 15.4$$

$\mathbf{TON_{Cu}}$ (15 min):

$$\mathrm{TON_{Cu,15min}} = \frac{4.48105 \times 10^{-4}}{1.27 \times 10^{-4}} = 3.53$$

Constant rate approximation:

$\mathbf{TON_{Pd}}$ (18 hours):

$$\mathrm{TON_{Pd,18h}} = 61.6 \times 18 = 1478.4$$

$\mathbf{TON_{Cu}}$ (18 hours):

$$\mathrm{TON_{Cu,18h}} = 15.4 \times 18 = 338.4$$

Time-on-Stream integration:

$$\int_{1}^{18} \left(\frac{r_i}{r_0} \times \Delta \mathrm{t}\right) = 15.742 \quad \Delta t = 1$$

$\mathbf{TON_{Pd}}$ (18 hours):

$$\mathrm{TON_{Pd,18h}} = 61.6 \times 15.742 = 969.7$$

$\mathbf{TON_{Cu}}$ (18 hours):

$$\mathrm{TON_{Cu,18h}} = 15.4 \times 15.742 = 221.9$$

Table S14. Performance metrics of the continuous-flow catalytic system, summarizing reaction yield, product formation and mass rates, molar and mass-based space–time yields (STY), catalyst productivity, liquid hourly space velocity (LHSV), and residence time under the applied operating conditions.

Metric	Formula	Value
Yield to product	$\mathrm{Y_P} = \frac{\dot{\mathrm{n}}_{\mathrm{Product}}}{\dot{\mathrm{n}}_{\mathrm{Iodobenzene}}}$	91.45%
Product rate	$\dot{\mathrm{n}}_{\mathrm{Product}}$	1.79242 mmol/h
Product mass rate	$\dot{\mathrm{m}}_{\mathrm{P}} = \dot{\mathrm{n}}_{\mathrm{P}} \times \mathrm{MW}$	0.31946 g/h
Reactor volume	$\mathrm{V_R}$	0.80 mL
STY (molar)	$\mathrm{STY} = \frac{\dot{\mathrm{n}}_{\mathrm{Product}}}{\mathrm{V_R}}$	2.2405 mol·L^{-1}·h^{-1}
STY (mass)	$\mathrm{STY_m} = \mathrm{STY} \times \mathrm{MW}$	399.33 g· L^{-1}·h^{-1}

Catalyst productivity	$\frac{\dot{n}_P}{m_{cat}}$	35.85 mmol·h^{-1}·g$_{cat}^{-1}$
Catalyst mass productivity	$\frac{\dot{m}_P}{m_{cat}}$	6.389 g· h^{-1}·g$_{cat}^{-1}$
LHSV	$\frac{Q_{Total}}{V_R}$	7.5 h^{-1}
Residence time	$\tau = \frac{V_R}{Q_{Total}}$	8 min
TOF (Pd basis)	$\frac{\dot{n}_P}{n_{Pd}}$	61.6 h^{-1}
TOF (Cu basis)	$\frac{\dot{n}_P}{n_{Cu}}$	14.1 h^{-1}
TON (Pd, 15 min)	$\frac{n_P}{n_{Pd}}$	15.4
TON (Cu, 15 min)	$\frac{n_P}{n_{Cu}}$	3.53
TON (Pd, 18 h, constant-rate)	$TON_{Pd,18h} = TOF_{Pd} \times t$	1478.4
TON (Cu, 18 h, constant-rate)	$TON_{Cu,18h} = TOF_{Cu} \times t$	338.4
TON (Pd, 18 h, time-resolved)	$TON_{Pd,18h} = TOF_{Pd} \times t$	969.7
TON (Cu, 18 h, time-resolved)	$TON_{Cu,18h} = TOF_{Cu} \times t$	221.9

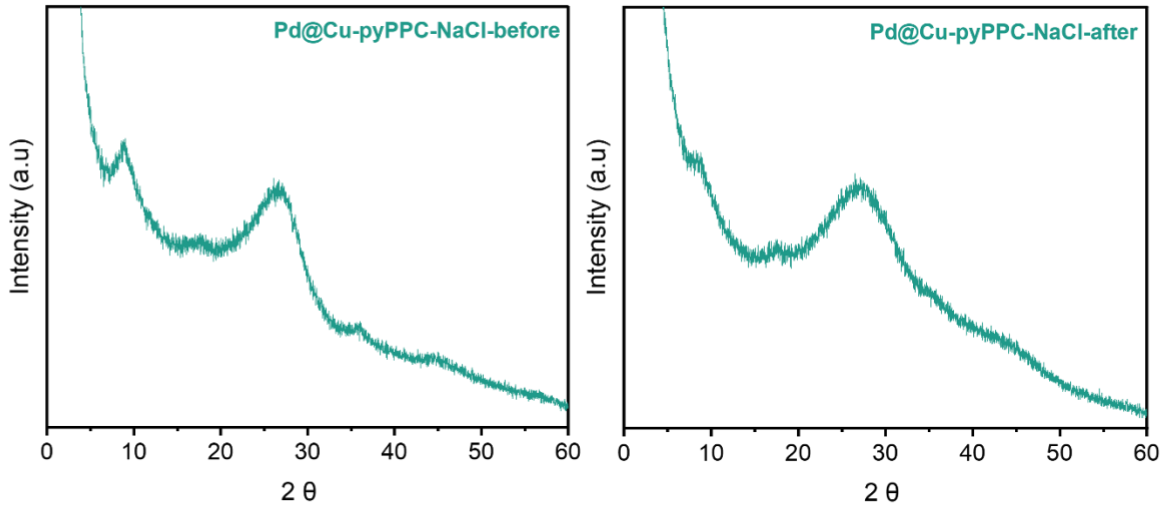

Figure S4. PXRD patterns of Pd@Cu-pyPPC-NaCl before and after 10 cycles.

References

(1) Witkowski, T. G.; Sebastiao, E.; Gabidullin, B.; Hu, A.; Zhang, F.; Murugesu, M. 2,3,5,6-Tetra(1H-Tetrazol-5-Yl)Pyrazine: A Thermally Stable Nitrogen-Rich Energetic Material. *ACS Appl. Energy Mater.* **2018**, *1* (2), 589–593. https://doi.org/10.1021/acsaem.7b00138.

(2) Clark, A. H.; Imbao, J.; Frahm, R.; Nachtegaal, M.; Clark, A. H.; Imbao, J.; Frahm, R.; Nachtegaal, M. ProQEXAFS: A Highly Optimized Parallelized Rapid Processing Software for QEXAFS Data. *urn:issn:1600-5775 27* (2). https://doi.org/10.1107/S1600577519017053.

(3) Newville, M.; Newville, M. Larch: An Analysis Package for XAFS and Related Spectroscopies. *J. Phys.: Conf. Ser.* **2013**, *430* (1). https://doi.org/10.1088/1742-6596/430/1/012007.

(4) J. J. Rehr; F. D. Vila; M. P. Prange; K. Jorissen; J J Kas. Parameter-Free Calculations of X-Ray Spectra with FEFF9. *Phys. Chem. Chem. Phys.* **2010**, *12* (21). https://doi.org/10.1039/B926434E.
